# Decoding pain through facial expressions: a study of patients with migraine

**DOI:** 10.1186/s10194-024-01742-1

**Published:** 2024-03-11

**Authors:** Wei-Ta Chen, Fu-Jung Hsiao, Gianluca Coppola, Shuu-Jiun Wang

**Affiliations:** 1 Brain Research Center, National Yang Ming Chiao Tung University, 155, Linong Street Sec 2, Taipei, 112 Taiwan; 2 School of Medicine, National Yang Ming Chiao Tung University, Taipei, Taiwan; 3 Department of Neurology, Neurological Institute, Taipei Veterans General Hospital, Taipei, Taiwan; 4 Department of Neurology, Keelung Hospital, Ministry of Health and Welfare, Keelung, Taiwan; 5 Department of Medico-Surgical Sciences and Biotechnologies, Sapienza University of Rome Polo Pontino, Latina, Italy

**Keywords:** Facial expression, Pain, Facial Action Coding System (FACS), Episodic migraine (EM), Chronic migraine (CM)

## Abstract

**Background:**

The present study used the Facial Action Coding System (FACS) to analyse changes in facial activities in individuals with migraine during resting conditions to determine the potential of facial expressions to convey information about pain during headache episodes.

**Methods:**

Facial activity was recorded in calm and resting conditions by using a camera for both healthy controls (HC) and patients with episodic migraine (EM) and chronic migraine (CM). The FACS was employed to analyse the collected facial images, and intensity scores for each of the 20 action units (AUs) representing expressions were generated. The groups and headache pain conditions were then examined for each AU.

**Results:**

The study involved 304 participants, that is, 46 HCs, 174 patients with EM, and 84 patients with CM. Elevated headache pain levels were associated with increased lid tightener activity and reduced mouth stretch. In the CM group, moderate to severe headache attacks exhibited decreased activation in the mouth stretch, alongside increased activation in the lid tightener, nose wrinkle, and cheek raiser, compared to mild headache attacks (all corrected *p* < 0.05). Notably, lid tightener activation was positively correlated with the Numeric Rating Scale (NRS) level of headache (*p* = 0.012). Moreover, the lip corner depressor was identified to be indicative of emotional depression severity (*p* < 0.001).

**Conclusion:**

Facial expressions, particularly lid tightener actions, served as inherent indicators of headache intensity in individuals with migraine, even during resting conditions. This indicates that the proposed approach holds promise for providing a subjective evaluation of headaches, offering the benefits of real-time assessment and convenience for patients with migraine.

**Supplementary Information:**

The online version contains supplementary material available at 10.1186/s10194-024-01742-1.

## Introduction

Migraine, a prevalent neurological disorder affecting more than one billion people globally and with a worldwide age-standardised prevalence of 14.4%, imposes significant disability [1]. Migraine is characterised by recurrent headache attacks accompanied by symptoms such as nausea, vomiting, photophobia, and phonophobia and has a considerable socioeconomic impact on affected individuals because it can result in substantial functional disability [2]. This impact is particularly pronounced when migraine progresses from episodic migraine (EM) to chronic migraine (CM), defined as an individual having more than 15 monthly headache days, with 8 days meeting the migraine diagnostic criteria. Effectively addressing the effects of migraines requires precise assessments of pain intensity during attacks, which can enable health-care professionals to make informed decisions about diagnosis and treatment. However, challenges related to the subjectivity of pain perception, the lack of biomarkers, variability in pain patterns, and inconsistencies in patient reporting considerably impede clinicians’ ability to complete real-time and reliable evaluations of pain.

In response to these challenges, researchers and clinicians have explored and established objective measures for assessing pain intensity or headache severity. By employing neuroimaging approaches involving brain recordings, studies have revealed connections between neural activities and pain levels. For example, Wager et al. (2013) employed a functional magnetic resonance imaging (fMRI)-based measure to predict pain intensity induced by noxious heat in healthy individuals and identified brain measures sensitive and specific to physical pain [3]. In another fMRI whole-brain volumes study, Marquand (2010) reported quantitative predictions of subjective pain intensity by using Gaussian process models [4]. Additionally, an electroencephalography study conducted by Nickel (2017) revealed cerebral representations of noxious stimulus intensity and pain intensity during painful heat stimulation [5]. In the context of patients with migraine, Bassez et al. (2022) reported that dynamic effective connectivity patterns of electroencephalography activities encode fluctuating pain intensity in patients with CM [6]. Furthermore, resting-state fMRI connectivity between specific regions was found to be correlated with pain intensity during acute migraine attacks [7]. However, Hoeppli et al. (2022) [8] suggested a dissociation between individual pain intensity and underlying brain activation, leading to the conclusion that fMRI may not be a reliable objective measure for inferring reported pain intensity. In our previous magnetoencephalographic study, our results indicated that individual pain sensitivity is associated with resting-state cortical oscillations in healthy individuals but not in patients with migraine [9]. Moreover, an fMRI study by Mayr et al. (2022) demonstrated that individually unique dynamics of cortical connectivity reflect the ongoing intensity of chronic pain [10]. No consensus has been arrived at with respect to cortical encoding of pain intensity in patients with pain disorders when neuroimaging techniques are employed. This may be because the relationship between pain intensity and neuroimaging findings is complex and multifaceted. Individual differences in pain perception and the influence of psychological factors can contribute to variability in neuroimaging results. Notably, in terms of their feasibility for point-of-care applications in clinics, neuroimaging techniques continue to have limitations, particularly because no quickly and conveniently identifiable characteristics have been discovered.

Analysing pain behaviours can enable assessment of individual pain intensity in patients with migraine. Such behaviours include facial expressions, verbal cues, body movements, alterations in postures or activity levels, and emotional responses [11]. In clinical settings, considering objectively measurable facial expressions in addition to subjective verbal expressions of pain may offer a more accurate understanding of a patient’s pain experience [12, 13]. Research on facial expressions in response to pain has identified specific movements associated with pain, such as lowering the brow, tightening the lids, raising the cheeks or fully closing the eyes, raising the upper lip, deepening the nasolabial fold, wrinkling the nose, and opening the lips and mouth to varying degrees. These facial expressions have consistently emerged across diverse experimental pain modalities [14–17] and various clinical pain conditions [18–20]. However, during physician visits, patients often mask their facial expressions and therefore appear calm; consequently, their expressions differ from those in experimental setups involving external noxious stimulation. In addition, the characteristics of facial expressions in patients with migraine during acute or chronic attacks remain unclear and warrant further investigation.

Pain is a complex experience encompassing sensory, affective-motivational, and cognitive dimensions. Facial expressions related to pain may reflect the amalgamation of individual perceptions, emotions, and cognitions. However, some facial expressions may result from emotional activation unrelated to pain. For instance, expressions conveying sadness or depression, such as raised inner corners of the eyebrows, loose eyelids, and downturned lip corners [21], have been observed in both healthy individuals and those with various diseases [22, 23]. Notably, emotional issues often coexist with pain disorders [24, 25]. The specific association between particular facial expressions and the emotional aspects of pain disorders remains uncertain.

We hypothesised that identifying specific elements of facial expression could offer supplementary insights that could aid clinicians in evaluating the current pain intensity of headaches, even during resting condition, with variations corresponding to different degrees of headache severity. Therefore, the present study investigated whether components of facial activities characterising facial expression could encode information about pain during headache attacks. Additionally, the study analysed the facial expressions in healthy controls (HCs) and patients with migraine to reveal potential differences associated with migraines.

## Materials and methods

### Participants

All participants were recruited from the headache clinic of Taipei Veterans General Hospital. Specifically, migraine patients were enrolled in the outpatient department by neurologists (WTC & SJW), while HCs actively participated in the study through advertisements or referrals from colleagues. The inclusion criteria were as follows: (1) all participants were between 20 and 60 years old, (2) they exhibited normal results on physical and neurological examinations, (3) patients with EM and CM were diagnosed according to the International Classification of Headache Disorders, Third Edition (ICHD-3) [26], and (4) HC participants rejected personal histories of migraine disorder or had experienced any significant pain condition over the previous year. However, given the high prevalence of tension-type headache or neck pain [27], mild non-migraine headaches were permitted in HC participants. The exclusion criteria included: (1) a history of systemic or major neurological diseases, (2) patients undergoing preventive treatment for migraine or diagnosed with medication overuse headache, (3) a history of substance abuse, and (4) participants currently undergoing Botulinum Toxin therapy. The hospital’s Institutional Review Board approved the study protocol (VGHTPE: IRB 2015-10-001BC), and all participants provided written informed consent before study commencement.

All participants were given semistructured questionnaires that were used to collect demographic information. On the day of the recording, the participants’ current headache pain intensity was assessed using a numerical rating scale (NRS) with endpoints ranging from 0 to 10, and their scores on the Hospital Anxiety and Depression Scale (HADS) [28] were recorded. The headache profiles for the patients with migraines were documented, and these profiles included the number of headache days per month and responses to the Migraine Disability Assessment (MIDAS) questionnaire, which assesses the extent of migraine-related disability [29]. Facial expression recording was conducted for each participant. For the patients experiencing headaches, the use of analgesics, triptans, or ergots was prohibited within 48 h before the recording.

### Experimental design

**Fig. 1:**
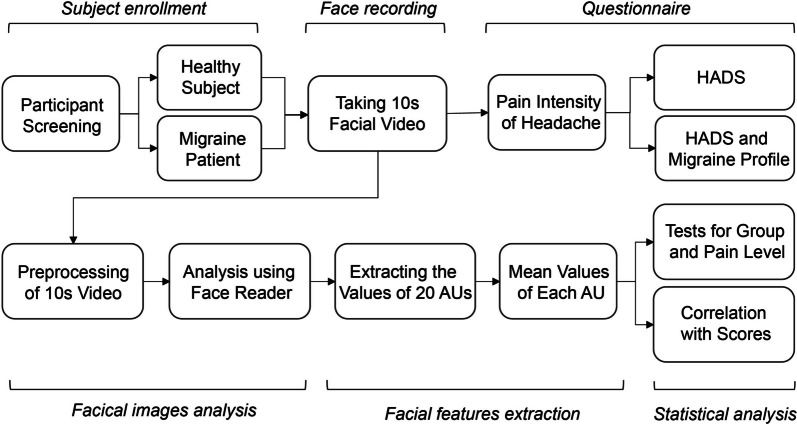
Pipeline of experimental procedure and face analysis

In the experimental procedure outlined in Fig. 1, attending physicians first screened the participants to determine their eligibility on the basis of the study’s inclusion and exclusion criteria. The participants were then categorised into either the HC group or a migraine group (EM or CM) on the basis of their self-reported statements and reevaluated after maintaining a headache diary for one month. Subsequently, face videos were recorded using the built-in camera of an iPad 7 (resolution: 1920 × 1080, frame rate: 30). The participants were positioned on a chair against a pure white wall background with adequate lighting and favourable contrast and brightness settings. The distance between the camera and the participant was 1 m, and the images of the upper body were capturing with the face cantered. To ensure that clear facial features were obtained for subsequent image analysis, any hair or objects potentially covering the face were removed. Throughout the 10-s recording, the participants were instructed to look directly at the camera and maintain a state of rest and calmness. Following the face video recording, the participants were asked to rate the current pain level of their headache by using the NRS. Subsequently, they completed the HADS questionnaire. Additional responses regarding their migraine profiles were obtained from the participants with migraine.

### Analysis of facial images

In the facial video preprocessing phase (Fig. 1), the initial 2 s and the final 2 s of each recording were omitted to minimise the influence of other emotional factors, such as tension or fatigue. Subsequently, we extracted a 5-s segment from each facial video (approximately 150 images per participant), ensuring the segments did not contain any face or head movement.

All facial images were imported into FaceReader software (Version 9, Noldus Information Technology, Netherlands) and coded using the Facial Action Coding System (FACS, https://www.noldus.com/applications/facial-action-coding-system) [30]. The analysis utilised a set of 20 action units, which represent facial expression and indicate the involvement of muscle activities (refer to supplementary Table 1 for details). The software first finds a person’s face, and then creates a 3D Active Appearance Model (AAM) [31] of a face. In the last stage, the AAM is used to compute scores of probability and intensity of facial expressions on a continuous scale from 0 to 1. In cases where the program detected insufficient image quality, the facial data were excluded from subsequent analysis. The FACS categorises facial movements on the basis of their appearance and deconstructs them into specific action units (AUs) along with temporal segments, which contribute to the overall expression. This technique enables objective description of facial expressions through identification of individual muscle activations. In this study, each facial image was used to compute intensity scores for each AU, with expressions rated on a continuous scale from 0 to 1. The analysis settings of the FaceReader software were as follows: (1) use of a set of 20 AUs (details in supplementary Table 1), (2) selection of the EastAsian face model, (3) a sampling rate for every image, and (4) no continuous calibration. After the analysis using FaceReader was completed, a facial expression matrix (20 AUs × 150 images) was obtained for each participant. Subsequently, the averaged value of each AU was calculated to characterise the facial expression for each participant.

### Statistics

On the basis of their NRS scores, the participants were categorised into three conditions: no pain (NP; NRS score: 0), mild pain (MP; NRS score: 1–3), and moderate to severe pain (SP; NRS score: 4–10). The demographics and clinical profiles were compared between groups (HC, EM, and CM) and conditions (NP, MP, and SP) by using chi-square tests or analysis of variance when appropriate. A permutation test, involving 10,000 permutations, was employed to analyse differences in the values of each AU for group and condition factors. A permutation test was also conducted to assess significance in the interaction of group and condition. Additionally, Pearson correlation analysis was employed to determine the correlation between AU values and clinical scores, including NRS and anxiety and depression scores. False discovery rate correction was applied for multiple comparisons, and a corrected *p* value of < 0.05 was considered statistically significant. Notably, the sample size was estimated through the use of G*Power 3.1 software [32]. Due to the absence of prior related research or established knowledge regarding effect size, a post-hoc power analysis was conducted to estimate the achieved power in the tests. A significance level (α) of 0.05 was selected, and the sample sizes for each group or condition were input into the G*Power software, employing nonparametric tests. All calculated powers exceeded 0.99, signifying ample sample size for this study.

## Results

### Demographic and clinical data

A total of 304 participant were recruited consecutively in this study, including 46 HCs, 186 patients with EM, and 72 patients with CM (Table 1). No significant differences were observed between the groups in terms of age (F = 1.73, *p* = 0.18) and sex (χ^2^ = 1.05, *p* = 0.59). Regarding NRS scores, the CM group exhibited higher scores than both the HC (*p* < 0.0001) and the EM (*p* < 0.0001) group did, whereas the EM group exhibited higher values than the HC group did (*p* < 0.001). Regarding psychometrics, the anxiety scores in the HC group were significantly lower than those in the EM (*p* < 0.001) and CM (*p* < 0.001) groups. Similarly, the HC group had lower depression scores than both the EM (*p* < 0.001) and the CM (*p* < 0.001) groups did. Moreover, the depression scores in the CM group were significantly higher than those in the EM group (*p* = 0.028). In terms of migraine profiles, as expected, the CM group had more headache days (*p* < 0.0001), and the MIDAS score was higher in the CM group than in the EM group (*p* = 0.007).

**Table 1:** Demographics and clinical scores of participants (mean ± std.)

	**HC**	**EM**	**CM**
***N***	46	186	72
***Demographics***			
Age (years)	31.4 ± 8.4	34.3 ± 9.5	34.4 ± 10.2
Sex	32F/14M	136F/50M	66F/16M
***Psychometrics***			
NRS	0.36 ± 0.97	1.49 ± 2.0	2.84 ± 2.4^*^
HADS_A	4.7 ± 3.4	7.6 ± 3.6	8.4 ± 3.8^$^
HADS_D	2.9 ± 2.2	5.3 ± 3.3	6.4 ± 4.3^#^
***Migraine profile***			
Headache days (/month)	-	6.6 ± 3.7	21.7 ± 6.5^η^
MIDAS	-	22.1 ± 27.9	35.3 ± 46.9^β^

*HC* Healthy control, *CM* Chronic migraine, *F* female, *M* male, *NRS* numerical rating scale, *HADS* Hospital anxiety and depression score, *A* Anxiety, *D* Depression, *MIDAS* Migraine disability assessment scores

^*^p < 0.01 (CM vs. HC & EM, EM vs. CM)

^$^*p* < 0.001 (HC vs. CM & EM)

^#^*p* < 0.05 (CM vs. HC & EM, EM vs. CM)

^η^*p* < 0.0001 (CM vs. EM)

^β^*p* < 0.01 (CM vs. EM)

Regarding pain conditions (Table 2), the participants were categorised into NP (137), MP (115), and SP (52) conditions. No significant differences were observed between the conditions in terms of age and sex (all *p* > 0.05). The anxiety scores were higher in the MP (*p* < 0.001) and SP (*p* < 0.0001) conditions than in the NP condition. Additionally, the depression scores in the SP condition were higher than those in the NP condition (*p* = 0.003).

**Table 2:** Demographics and clinical scores for pain conditions (mean ± std.)

	**NP**	**MP**	**SP**
***N***	137	115	52
HC	39	7	0
EM	85	72	29
CM	13	37	22
***Demographics***			
Age (years)	32.5 ± 8.8	34.7 ± 9.3	36.3 ± 11.6
Sex	92F/45M	91F/24M	41F/11M
***Psychometrics***			
NRS	0 ± 0	1.8 ± 0.9^&^	5.6 ± 1.5^*^
HADS_A	6.3 ± 3.5	7.8 ± 3.8^%^	8.9 ± 4.0^$^
HADS_D	4.5 ± 2.9	5.6 ± 3.6	6.3 ± 4.7^#^

*NP* no pain condition, *MP* mild pain condition, *SP* moderate to severe pain condition; *HC* Healthy control, *CM* Chronic migraine, *F* female, *M* male, *NRS* numerical rating scale, *HADS* Hospital anxiety and depression score, *A* Anxiety, *D* Depression

^&^*p* < 0.0001 (MP vs. NP)

^*^p < 0.0001 (SP vs. NP)

^%^*p* < 0.05 (MP vs. NP)

^$^*p* < 0.05 (SP vs. NP)

^#^*p* < 0.05 (SP vs. NP)

### Differences in facial features between groups and conditions

**Fig. 2:**
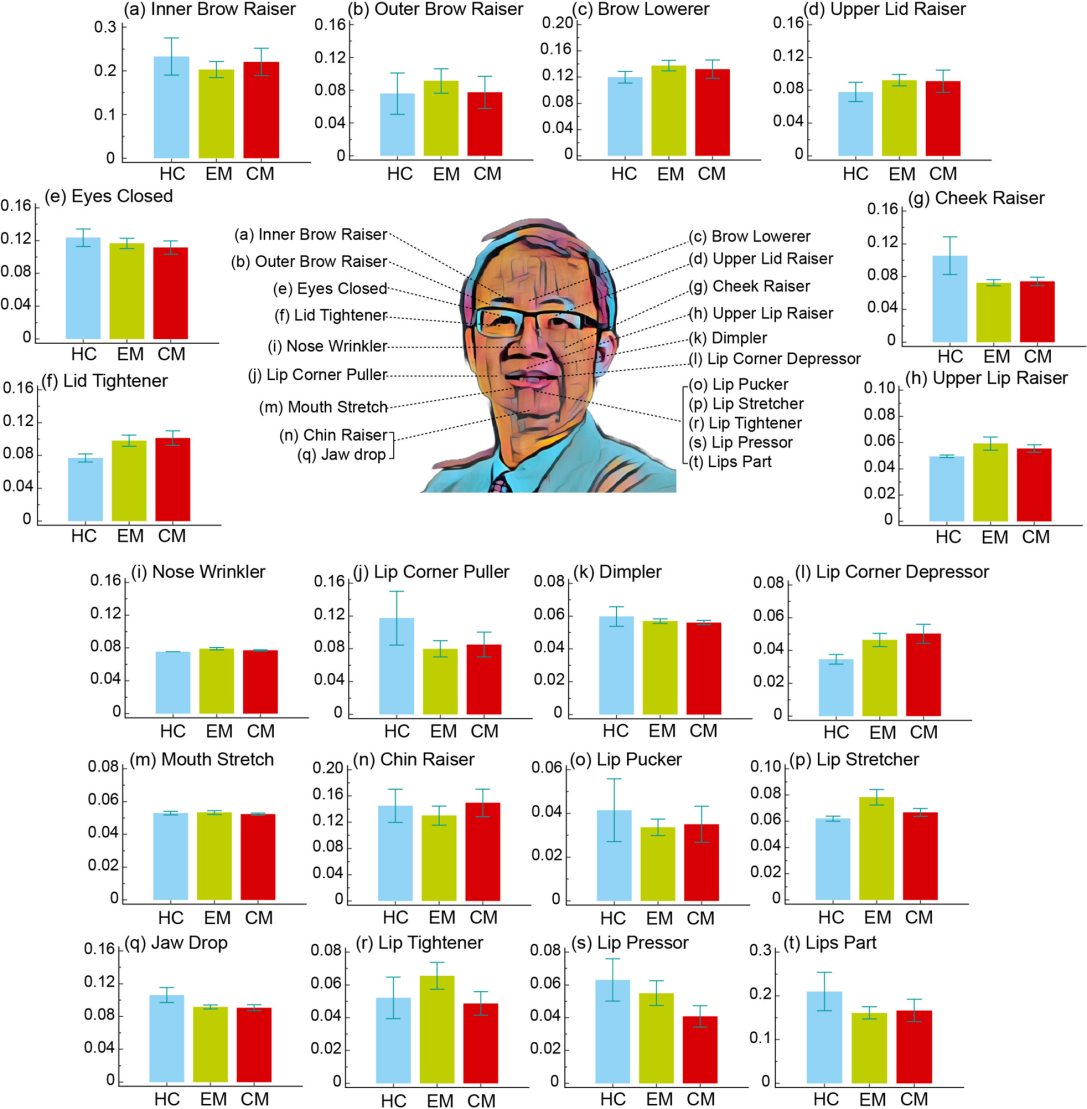
Activation differences of 20 facial muscle activities between groups. HC, healthy control; EM, episodic migraine; CM, chronic migraine

**Fig. 3:**
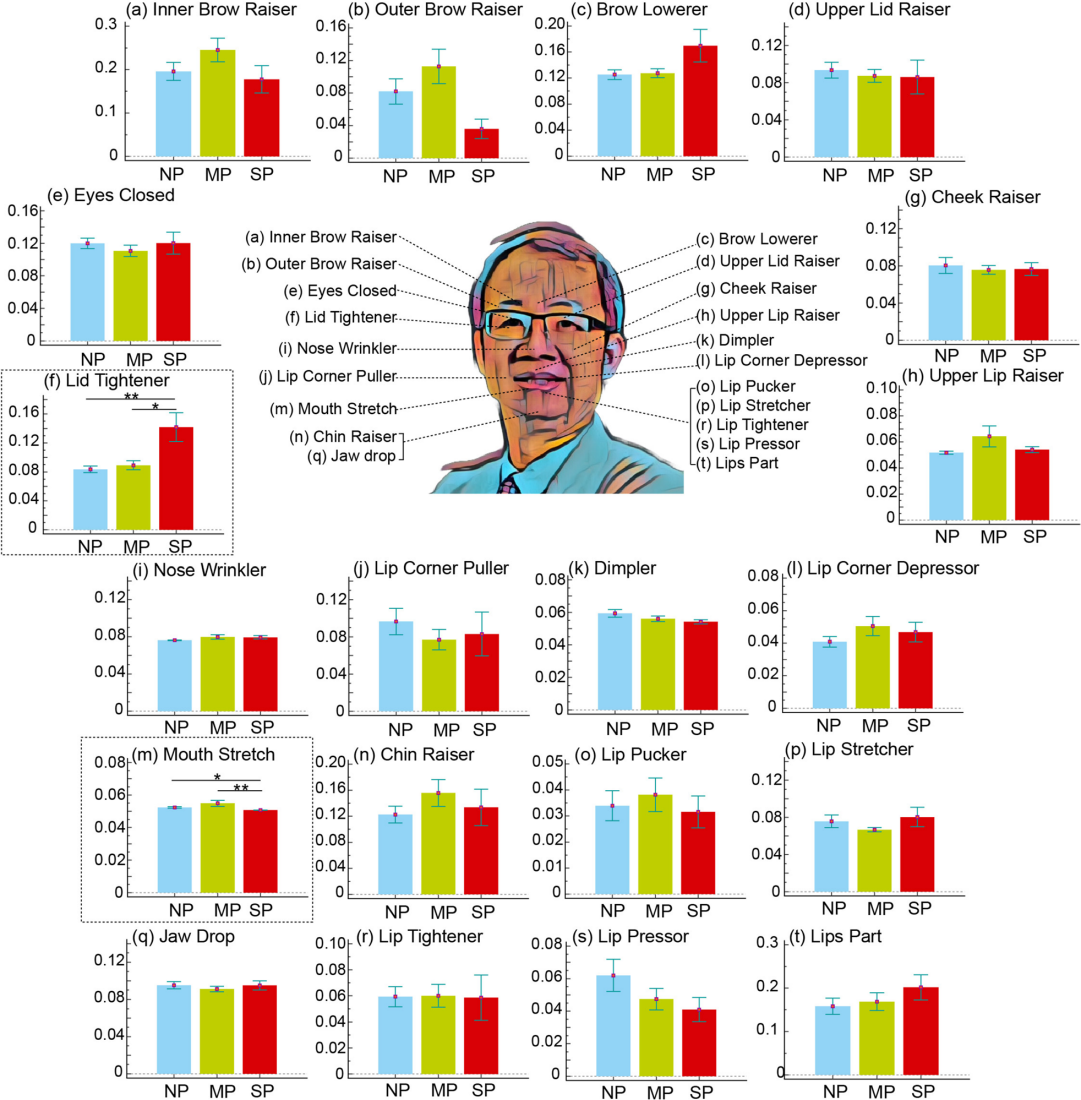
Activation differences of 20 facial muscle activities between pain conditions. NP, no pain; MP, mild pain; SP, moderate to severe pain. *, *p* < 0.05; **, *p* < 0.01

**Fig. 4:**
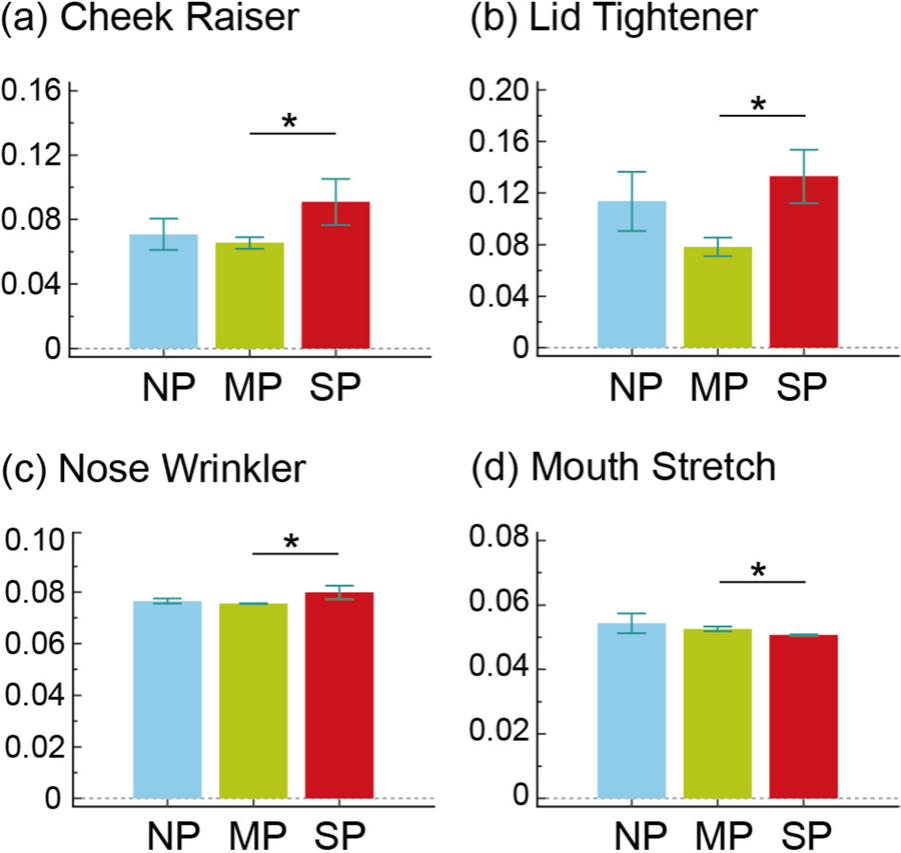
In patients with chronic migraine, prominent differences of facial muscle activities between pain conditions. NP, no pain; MP, mild pain; SP, moderate to severe pain. *, *p* < 0.05

Regarding the group factor (Fig. 2), all AU values were comparable (all corrected *p* > 0.05), indicating that facial expression in a state of rest and calmness did not significantly vary due to the presence of migraine and its chronification. However, regarding the pain condition factor (Fig. 3), significant differences were observed in two AUs, that is, the lid tightener and mouth stretch. This indicated increased activation of lid tightener in the SP condition compared with in the NP (corrected *p* < 0.01) and MP (corrected *p* < 0.05) conditions as well as reduced activation of mouth stretch in the SP condition than in the NP (corrected *p* < 0.05) and MP (corrected *p* < 0.01) conditions. Among the patients with CM, the intensity of AUs in the SP condition significantly differed from that in the MP condition (Fig. 4), with reduced activation noted for mouth stretch (corrected *p* < 0.05) and increased activation noted for lid tightener (corrected *p* < 0.05), nose wrinkle (corrected *p* < 0.05), and cheek raiser (corrected *p* < 0.05). However, no significant changes were noted between pain conditions in the HC and EM groups.

### Correlation among facial features and clinical scores

**Fig. 5:**
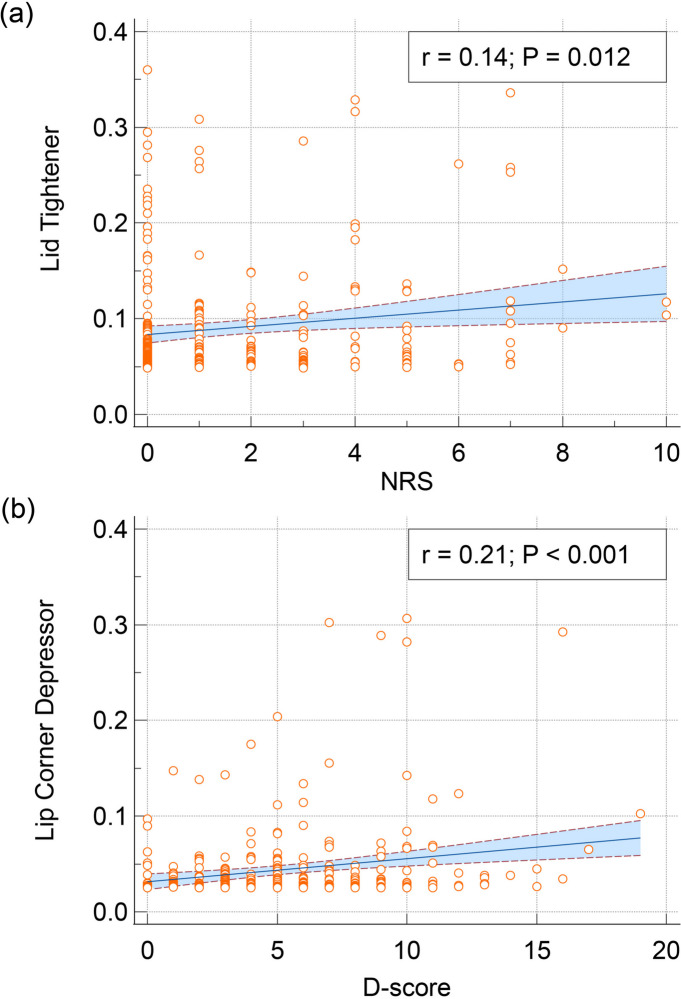
**a** Correlation between lid tightener activation and numerical rating scale (NRS) of headache **b** Correlation between lip corner depressor activation and depression score (D-score)

For all participants, a positive correlation was noted between the activation of lid tightener and NRS scores (*r* = 0.14, *p* = 0.012, upper part in Fig. 5), indicating that a more pronounced tightening of the eyelid was associated with greater perceived headache pain severity. Additionally, depression scores were associated with lip corner depressor activation (*r* = 0.21, *p* < 0.001, lower part in Fig. 5), indicating that the activation of the facial muscle depressor anguli oris gradually increased as the level of depression increased.

## Discussion

In this study, we explored differences in facial expressions among patients with migraine and analysed the relationship between these facial expressions and headache pain intensity. Our approach involved capturing facial videos while participants maintained a calm face in resting conditions. Facial expressions were assessed using 20 AUs, with each representing specific muscle activations. Notably, our findings revealed that the facial expressions in patients with migraine (those with EM or CM) did not significantly differ from those of HCs. An increased level of headache pain was associated with increased activation of lid tightener and reduced activation of mouth stretch among participants. In the patients with CM, moderate to severe headache attacks were characterised by reduced activation of mouth stretch, accompanied by increased activation of lid tightener, nose wrinkle, and cheek raiser. Notably, the activation of lid tightener exhibited a positive correlation with the headache level, as assessed using NRS scores. Additionally, lip corner depressor emerged as an indicator of the degree of emotional depression. These findings underscore the potential for real-time and reliable clinical evaluation of pain in patients with migraine on the basis of facial expressions.

### Facial expressions related to headache pain intensity

The intensity of lid tightener and mouth stretch in the calm facial expression was correlated with the current severity of headaches in patients with migraine. These findings are in line with those of previous research, which has consistently demonstrated notable changes in facial expressions in response to external pain stimuli [14–17] and across various pain disorders [18–20]. These alterations include actions such as lowering the brow, tightening the lids, elevating the cheeks or fully closing the eyes, lifting the upper lip, deepening the nasolabial fold, wrinkling the nose, and opening the lips and mouth to varying degrees. Notably, in contrast to earlier experimental setups, that of this study was established with a focus on capturing calm faces during resting conditions, making it suitable for point-of-care applications in clinics without any noxious stimulation. In this study, distinct facial features in patients with migraine emerged as indicative of muscular activation changes during rest. Lid tightener was identified as a marker of the level of headache pain intensity and was correlated with the NRS score for headache severity. Lid tightener is regulated by the orbicularis oculi, a muscle activated by the trigeminal nerve. Moreover, bright light could activate nociceptive neurons in superficial laminae of trigeminal subnucleus caudalis driven by a reflex circuit [33]. In the context of migraines, the trigeminal nerve plays a pivotal role in transmitting pain signals from the brain to the face, the site of headache attacks [34]. Notably, in patients with CM, occipital nerve stimulation was demonstrated to markedly reduce the orbicularis oculi reflex. This finding indicates that direct counteraction of trigeminally mediated central sensitisation mitigates the effects of aversive peripheral stimulation [35]. We suggest that heightened lid tightener intensity may reduce light input triggering the trigeminal nociceptive pathway. Consequently, the contraction of the orbicularis oculi proportionally reflected the intensity of the headache attack. This effect persists even when patients with migraine attempt to maintain a calm facial expression, as recorded in our study. Therefore, evaluation of lid tightener through facial images could be used in clinical assessments of headache pain with real-time and reliable characteristics.

### No significant differences in facial expression between groups

No significant differences in the activation of the 20 AUs were observed during calm facial expressions among the HCs, patients with EM, and patients with CM. This indicates that patients with migraine, even those experiencing nearly daily headaches, as seen in CM, did not exhibit distinct facial expressions during resting states. However, distinct features in facial expressions were observed during moderate to severe headache attacks, providing a potential avenue for identification in such instances. Consistent with previous studies, in the present study, the patients with migraine did not exhibit significantly different absolute electromyography (EMG) levels in the frontal, temporal, or corrugator muscles, nor did they exhibit distinct facial muscle responses to stress compared with those of the HCs [36]. Similar results were observed over the trapezius muscle [37]. In another study, EMG responses in patients with headache, specifically in the trapezius, neck (splenius), temporalis, and frontalis areas, did not significantly differ from controls [38]. Moreover, no notable correlation was observed between EMG responses for facial and head muscles and individuals’ pain reactions [38]. These findings indicate that muscle activities over the face, neck, and head areas remain within normal ranges in patients with migraine. Taken together, the observed changes in facial activities in the present study may be a consequence of the headache attack rather than represent a specific feature unique to migraines.

### Differences in facial expression between pain conditions in patients with CM

Significant alterations in facial expressions were observed in the patients with CM in SP conditions. These patients exhibited heightened activation of facial muscles, including lid tightener, nose wrinkle, and cheek raiser, during moderate to severe headache attacks and reduced activation of the muscles responsible for mouth stretch. Notably, these expressions are generally associated with emotions such as fear, disgust, and anger. Conversely, the patients with EM did not exhibit significant changes in the activation intensity of these facial features during headache attacks. Several factors may explain these contrasting findings. First, the frequent headache attacks experienced by patients with CM have been associated with both central hyperexcitability [39–41] and peripheral dysfunction [42], potentially contributing to pronounced changes in facial activities even during calm or resting conditions. Second, emotional disturbances were more prevalent in the patients with CM than in those with EM, which may account for their sustained alterations in facial activities. Finally, symptoms such as photophobia, phonophobia, and allodynia, which are commonly experienced by patients with CM, particularly during headache attacks [43], might have played a role in the observed altered facial expressions in SP conditions.

Significantly, the facial distinctions noted in patients with CM were evident between the SP and MP conditions rather than between the SP and NP conditions. A study indicated that even during the interictal (pain-free) phase, patients with migraine may experience disease-related symptoms such as cutaneous allodynia, cognitive impairment, photophobia, and reduced health-related quality of life [44]. Moreover, emotional effects, including those from anger, depression, anxiety, and hopelessness, were reported during this phase [45]. These findings indicate that facial activities in the NP condition may undergo alterations similar to those observed in the SP condition in patients with CM. However, in the current study, the patients with migraine experiencing MP, which has fewer emotional effects, exhibited fewer alterations in facial activities. Consequently, the averaged activation values within each condition exhibited a descending or increasing order of MP, NP, and SP for these five facial features.

### Depression facial characteristics involving lip corner depressor

Depression is a prevalent comorbidity in migraine, particularly in CM [46, 47]. In this study, the lip corner depressor was associated with the level of emotional depression. Facial expressions conveying sadness or depression, such as raised inner corners of the eyebrows, loose eyelids, and downturned lip corners, were observed [21]. These expressions have been observed in both healthy individuals and patients with various diseases [22, 23]. Notably, Hawk et al. (2012) reported a significant association between lip corner depressor activity and expressions of sadness [48]. However, the complex interactions between headache attacks and emotional depression and their effects on facial expressions remain unclear. This study, which captured calm faces, revealed distinct facial activities associated with headache severity (lid tightener) and emotional depression (lip corner depressor). We hypothesise that objectively evaluating the impact of headaches or depression on patients with migraine can be facilitated through analysis of facial expressions.

## Limitations

This study has several limitations that warrant consideration. First, the observed association between lid tightener facial activities and headache pain intensity may be specific to patients with migraine. Further investigation is required to determine whether this particular facial feature, observed in calm faces, is also indicative of or is the same for pain associated with other disorders. Second, due to the cross-sectional design of this study, facial features could not be validated individually on different days with varying headache intensities or during periods without a headache attack. Third, during facial video recording, the participants were instructed to maintain a calm facial expression in a resting condition. Given that patients with migraine experience altered pain perception and complex emotional problems [39, 49], to understand the influence of these two central processes on facial expression characteristics requires additional studies with more sophisticated designs. Moreover, this study assessed the averaged intensity of each AU, which may have led to specific facial expressions, such as that of apathy, which is frequent exhibited by individuals with Alzheimer’s disease, being overlooked [50]. Future investigations should consider differences in the intensity of each facial activity as a parameter. Finally, this study’s finding of a lack of discernible differences in facial activities between groups may be attributable to the experimental setup involving the resting condition during facial video capture. Given that previous findings indicate altered cortical activation in patients with CM during sensory or nociceptive processing [39, 51], additional research exploring the influence of stimulation on changes in facial activities could provide a more comprehensive understanding of this issue.

## Conclusion

In the present study, facial expressions, particularly lid tightener actions, reflected the intensity of headaches in the patients with migraine, even during resting conditions. Moreover, the level of depression could be inferred from facial muscle activities. This approach to assessing patients with migraine can enable subjective evaluation of headaches regarding the pain intensity and emotional problems. Furthermore, it can provide the advantages of real-time assessment and convenience through a 10-s facial video recording using a digital camera (preferably with an image resolution greater than 1920 × 1080) and approximately 5 min of facial expression analysis using FACS (FaceReader software). Further studies validating this approach across other chronic pain disorders are warranted to confirm its reliability and specificity.

### Supplementary Information

**Supplementary Material 1.**

## Data Availability

Derived data supporting the findings of this study are available on request from the corresponding authors.
