# Supplementary material for: Decoding pain through facial expressions: a study of patients with migraine
Source: J Headache Pain. 2024 Mar 11;25(1):33. doi: 10.1186/s10194-024-01742-1 (PMC10926654; doi:10.1186/s10194-024-01742-1)
Supplement: Supplementary file 1 — Supplementary Material 1. [file 10194_2024_1742_MOESM1_ESM.docx]

Supplementary Table 1 Facial Action Units

| No. | Action Unit | Item description | Muscular basis |
| --- | --- | --- | --- |
| 1 | 01 | Inner Brow Raiser | Frontalis |
| 2 | 02 | Outer Brow Raiser | Frontalis |
| 3 | 04 | Brow Lowerer | Depressor glabellae, Depressor supercilii, and Corrugator supercilii |
| 4 | 05 | Upper Lid Raiser | Levator palpebrae superioris, and Superior tarsal muscle |
| 5 | 06 | Cheek Raiser | Orbicularis oculi |
| 6 | 07 | Lid Tightener | Orbicularis oculi |
| 7 | 09 | Nose Wrinkler | Levator labii superioris alaeque nasi |
| 8 | 10 | Upper Lip Raiser | Levator labii superioris, Caput infraorbitalis |
| 9 | 12 | Lip Corner Puller | Zygomaticus major. |
| 10 | 14 | Dimpler | Buccinator |
| 11 | 15 | Lip Corner Depressor | Depressor anguli oris |
| 12 | 17 | Chin Raiser | Mentalis |
| 13 | 18 | Lip Pucker | Incisivii labii superioris and Incisivii labii inferioris |
| 14 | 20 | Lip Stretcher | Risorius w/ platysma |
| 15 | 23 | Lip Tightener | Orbicularis oris |
| 16 | 24 | Lip Pressor | Orbicularis oris |
| 17 | 25 | Lips Part | Depressor labii inferioris, or Mentalis or Orbicularis oris |
| 18 | 26 | Jaw Drop | Masseter, Temporalis and Internal pterygoid |
| 19 | 27 | Mouth Stretch | Pterygoids and Digastric |
| 20 | 43 | Eyes Closed | Levator palpebrae superioris |

From: Facial action coding system, FaceReader, Noldus Information Technology
